# Paving Pathways: shaping the Public Health workforce through tertiary education

**DOI:** 10.1186/1743-8462-7-2

**Published:** 2010-01-03

**Authors:** Catherine M Bennett, Kathleen Lilley, Heather Yeatman, Elizabeth Parker, Elizabeth Geelhoed, Elizabeth G Hanna, Priscilla Robinson

**Affiliations:** 1 School of Health and Social Development Deakin University, 221 Burwood Highway, Burwood, Australia; 2 Melbourne School of Population Health, The University of Melbourne, Australia; 3 School of Public Health, Griffith University, Australia; 4 School of Health Sciences, University of Wollongong, Australia; 5 School of Public Health, Queensland University of Technology, Australia; 6 School of Population Health, The University of Western Australia, Australia; 7 Public Health Association of Australia and National Centre for Epidemiology and Population Health, The Australian National University, Australia; 8 School of Health Sciences, La Trobe University, Australia

## Abstract

Public health educational pathways in Australia have traditionally been the province of Universities, with the Master of Public Health (MPH) recognised as the flagship professional entry program. Public health education also occurs within the fellowship training of the Faculty of Public Health Medicine, but within Australia this remains confined to medical graduates. In recent years, however, we have seen a proliferation of undergraduate degrees as well as an increasing public health presence in the Vocational Education and Training (VET) sector.

Following the 2007 Australian Federal election, the new Labour government brought with it a refreshing commitment to a more inclusive and strategic style of government. An important example of this was the 2020 visioning process that identified key issues of public health concern, including an acknowledgment that it was unacceptable to allocate less than 2% of the health budget towards disease prevention. This led to the recommendation for the establishment of a national preventive health agency (Australia: the healthiest country by 2020 National Preventative Health Strategy, Prepared by the Preventative Health Taskforce 2009).

The focus on disease prevention places a spotlight on the workforce that will be required to deliver the new investment in health prevention, and also on the role of public health education in developing and upskilling the workforce. It is therefore timely to reflect on trends, challenges and opportunities from a tertiary sector perspective. Is it more desirable to focus education efforts on selected lead issues such as the "obesity epidemic", climate change, Indigenous health and so on, or on the underlying theory and skills that build a flexible workforce capable of responding to a range of health challenges? Or should we aspire to both?

This paper presents some of the key discussion points from 2008 - 2009 of the Public Health Educational Pathways workshops and working group of the Australian Network of Public Health Institutions. We highlight some of the competing tensions in public health tertiary education, their impact on public health training programs, and the educational pathways that are needed to grow, shape and prepare the public health workforce for future challenges.

## Introduction

### The changing context for public health education

There are important changes occurring in the tertiary education landscape that provide a new context for discussion on directions and challenges in public health tertiary education. There is continuing pressure on universities to be less dependent on government funding, and associated pressures to increase the number of international fee-paying places. International students, who make up a considerable proportion of public health students at some institutions, often have different educational backgrounds and needs. They tend to fall into two distinct groups: either they are training to deal with different public health issues in their home countries when they return; or they are focussed on using public health training as a vehicle to start new lives away from their home countries.

Undoubtedly, competent international students add to the learning environment, particularly for local students interested in developing an international health understanding and skill set. However there is a real counterbalancing risk at the institutional level. Income-driven student recruitment policies can distract from the provision of education with a clear focus on what is in the best interests for public health in Australia.

Universities are also being encouraged to consider restructuring teaching programs and to reduce the length of postgraduate coursework award programs. Some are introducing articulated undergraduate - masters degrees where the higher degree is required for professional registration; for instance psychology, occupational therapy and dietetics at La Trobe University. Others are moving all professional entry degrees to graduate level, as seen for example with the introduction of the Melbourne Model at The University of Melbourne. All these changes impose pressure on degrees that have traditionally had a strong student base drawn from undergraduate-trained health and allied health professionals, and command new thinking about public health coursework and research educational pathways.

Health promotion and illness prevention are increasingly being recognised as vital to the wellbeing of the whole Australian population and essential for an economically sustainable health system, gaining traction, for example through Australia's 2020 summit resolutions and recommendations. The 2020 resolutions and recommendations together with the establishment of the National Preventive Health Agency reinforce the need for a strong, capable public health workforce to deliver the promised intensified focus on prevention and health promotion, and to characterise and address the major determinants of ill health and poor health outcomes.

The projected demographic shift towards an ageing population with decreasing number of young people expected to enter the health workforce over the next decade [1] will challenge our ability to meet community expectations of service delivery. Given the influence on public health outcomes of policies and actions emanating outside of the health system [2,3] it is evident that public health education should be available and accessible to professionals both within and outside the public health sector, not just to those with a clinical background or within traditional public health roles. We must therefore develop strategies to both increase public health recruitment, and to both broaden and deepen public health knowledge and skills in the wider health workforce.

Subsequent to the 1986 Kerr-White recommendations [4] the Commonwealth actively supported public health education. A workforce survey [5] was completed in the mid-1990s and the Public Health Education and Research Program (PHERP) was introduced. Since its establishment, PHERP has provided ongoing support to five state-based university consortia, four national and special focus centres and 41 innovation projects, as well as several workforce development projects such as the Masters of Applied Epidemiology program, a Biostatistics Collaboration, and a Public Health Registrars program. In 2001 the total program increased to $55 million per annum, an investment that no doubt increased the capacity for member institutions to build and deliver public health education programs. It has yet to be seen what impact the closing of the PHERP initiative after 2010 will have on post-graduate public health education in Australia.

Prior to the 2005 PHERP Phase III Review, the Australian Network of Academic Public Health Institutions (ANAPHI) produced a monograph of case studies, 'Building Capacity to Improve Public Health in Australia: Case Studies of Academic Engagement [6]. The monograph highlighted research, policy engagement and educational programs in Australia's universities that had demonstrated how academic public health institutions have contributed to improving public health capacity in Australia. Public health success stories included the response to SARS, advances in Indigenous health and the prevention and management of chronic diseases. The case studies highlighted the contribution of PHERP funding and its impact on the growth in public health capacity and improvements in the education of the public health workforce, particularly through Master of Public Health programs.

The National Health and Hospital Reform Commission Report (2009), recommends adoption of a competency-based framework as part of broad teaching and learning curricula for all health professionals. Public health has yet to identify a role in this agenda, and it is also unclear what role public health will play in the recommendation to establish a national clinical education and training agency.

The 2005 review of PHERP [7] also precipitated the establishment of minimum standards in public health competencies for graduates, aimed at MPH programs in particular. Subsequent discussion has centred on public health workforce needs, public health graduate competencies and the emerging definition and role of graduate attributes [8]. The draft core competencies for MPH graduates were completed in August 2009 and at the time of writing are in the process of ratification by public health education providers across Australia.

Major changes are also afoot internationally. The MPH is generally considered to be an internationally transportable degree, but this might not continue to be the case. The Bologna Process [9,10] is a European initiative designed to standardise certificated courses throughout the European Union (EU). As a part of this process, Master of Public Health courses taught within the EU will soon be accredited. Registration of the public health workforce will follow and hence will be subject to regulation [11]. Australia was one of four non-EU countries to be a signatory to the Bologna Agreement, but has apparently not remained engaged in these developments. The United States has taken a different pathway [12] and has developed an accreditation process linked to continuing professional development for the purposes of public health workforce regulation [13].

## Discussion

### What is a public health professional?

Public Health graduates have taken a population health perspective into a range of different employment settings; from specialist public health research and practice, to management and planning in health services, and clinical practice with a population orientation. The MPH and other specialist public health degrees build capacity in, and strengthen, evidence-based public health practice. Public health education also brings an evidence-based population health oriented approach to policy making and management at all levels and in all sectors of public health and health service delivery.

It differs from the clinical professions by focussing on what makes people sick and what keeps people well; that is, the determinants of health, and identifying which groups are vulnerable, and why this is the case. Public health then designs, implements and evaluates programs to maximise opportunities for health and reduce ill health. Public health education builds an understanding of what health means; a vital ingredient for health policy, from the local agency through to government level.

Public health graduates therefore provide significant enhancement to the public health and health services workforce. Yet, there remains substantial scope for further strengthening the values and perspective that public health training contributes.

The practice of public health encompasses many disciplines, and best practice relies on practitioners and researchers who have acquired interdisciplinary skills and perspectives. However, with the low priority traditionally given to public health within the health sector, as well as the dominance of the medical paradigm in Australia, relatively little attention has historically been given to a consideration of the definition of public health professionals, and therefore the potential sources and destinations of students, and their educational requirements. The (medical) faculties of Public Health Medicine in the United Kingdom, the USA and Australia, for example, have detailed the sets of competencies their graduates are expected to have, as have the public health training schemes provided through State Health Departments in New South Wales and, to a lesser degree, in Victoria. The Victorian Consortium for Public Health, together with the Australian Network of Public Health Institutions and Australian Government PHERP program, are currently engaged in public health employer and graduate surveys to assess current workforce educational needs.

The state government-based training schemes in NSW and Victoria have recently paired with universities in order to award a professional doctorate to those who successfully complete the specifically developed program (The University of New South Wales and Monash University respectively). Given the breadth of the concepts and skills covered in such training, as with the MPH, it will be a challenge to meet doctoral level competencies within such programs. Arguably, advanced educational programs are required to deliver high level knowledge and skills, either as a specialised stream within a two-year Masters or three-year doctoral program, or as a 'stand-alone' degree. However, the public health workforce is to a large degree characterised by their breadth of practice. This includes the wide span of contexts the practitioners work within, the disciplines they bridge within their routine practice, and the multi-professional teams they work within. Under this framework, the task of effectively assessing Doctoral or Masters level competency standards solely against traditional discipline-based benchmarks becomes problematic.

The public health workforce is largely employed in public sector and non-government organisations. Public health education programmes train the workforce in health research and policy development. Curricula also cover the implementation and evaluation of the outcomes of biomedical research and of policy by way of programme development. The theory and skills encompassed within public health training are therefore increasingly being recognised as important for a wide range of health professionals. Until the 1980s, public health comprised only a very small component of undergraduate medical and health sciences program content. Widening recognition of the value of public health perspectives for health care practice has driven a significant shift, and there are now many examples where public health is integrated into core undergraduate clinical health sciences training.

In more recent times, some Universities have perceived a need to develop undergraduate programs for entry-level public health practitioners, firstly for Australian health promotion professionals, and more recently with the introduction of undergraduate programmes specifically for public health practitioners. There are approximately 10 undergraduate public health programs offered in Australia [14]. In Queensland, health promotion and public health professionals including epidemiologists are now recognised under the Health Practitioner stream of the Queensland Health workforce. The Department of Health and Ageing have also provided financial support to the vocational education and training (VET) sector to develop training packages at the Certificate and Diploma level in population health.

These certificate level and tertiary undergraduate programs have to meet the challenge of educating for a professional group that is not well-defined because it does not have a clearly recognised professional identity. It may be easier to provide education for selected public health roles (for example programs to provide for the public health nutrition workforce in Queensland or health promotion roles more widely across Australia) than for a 'public health officer'; a term currently only used in New South Wales for their post-MPH-graduate trainees.

### Public health student profile

Whilst the MPH is globally recognised as the professional entry degree in public health, it cannot, and should not, be expected to deliver both a foundation in core public health skills and high-level specific skills training in more than one skill or discipline area. This disjunct will become even further pronounced as the background of students becomes more diverse and increasingly removed from the traditional clinical feeder pathways. Specialised award and non-award courses at different academic levels are an essential consideration in a comprehensive public health capacity building plan. These must also encompass pathways for continuing professional development, and research training opportunities.

Alongside the introduction of more specialised programs, we are seeing a trend towards more students embarking on public heath training earlier in their career; either undertaking an undergraduate degree in public health, or commencing an MPH or specialist public health degree soon after completion of their first degree. However many students in Australia still enter their public health training from the workforce, bringing considerable work experience from within the health sector, and continue to work whilst undertaking further study.

Universities need to respond to the challenges of developing and delivering programs suitable for this increasingly diverse student group, and continue to accommodate the different needs of the full-time employed students. For working students, access to public health education can be enhanced by employer support (time off for study, contribution to study fees) and the flexible delivery modes on offer (including intensive teaching blocks, distance education, on-line learning support and out-of-hours classes) and access to part time programs. Academia and industry must therefore work together to create the pathways that will rectify disincentives and encourage greater participation in further education by the public health workforce.

### Undergraduate versus postgraduate education

The growth in undergraduate public health education in Australia parallels trends internationally in the United States and in the Asia Pacific Region, for example in Vietnam and Thailand. In Australia, a range of undergraduate public health education programs exist, either as 'stand-alone' Bachelor of Health Science or Bachelor of Public Health awards or in combination with a wide variety of other degrees such as nursing, development studies and economics. The depth of learning and extent of skill development in the traditional public health sciences of epidemiology, biostatistics, research methods and public health practice varies considerably within these degrees. Some place particular emphasis on a combination of these sciences in addition to studies in health promotion and environmental health.

Are these degrees preparing 'beginning public health practitioners'? If so, what does this mean for the Master of Public Health, once the traditional domain of training for the entry-level public health practitioner? And what is the repertoire of skills and competencies that an undergraduate public health graduate brings to the workplace? Or are undergraduate health science degrees that focus on public health and health promotion to be viewed, not as professional entry, but as part of a 'liberal arts' background emphasising breadth of topics and analytical and critical thinking but with a focus on the health of populations? It is confusing for employers. How will prospective employers differentiate between potentially divergent skills levels in graduates across such varied training pathways? Will differences in training be consistent across educational institutions and States? Local understandings and skills required in Darwin may differ widely to those needed in Tasmania or urban Melbourne.

While there have been numerous projects mapping competencies for public health and health promotion via professional associations and some State governments in Australia and internationally, for example the Galway competencies for Health Promotion [15], these are broadly defined and not necessarily embedded in the distinctions between postgraduate and undergraduate levels. For example, the competency public health project commissioned through the quality framework of PHERP did not distinguish between these levels of education. The exercise of mapping curricula to such competency standards will ensure the public health sciences are appropriately embedded across undergraduate and post graduate curricula, but the expectations for undergraduates must be realistic.

There is no doubt, on the other hand, that public health career options should be made more visible to undergraduate students, whether specialising in public health or not, and Universities need to work with the public health professions and workforce to build the public profile of public health career pathways. Similarly, injection of core public health principles and concepts into undergraduate programs should be widespread, including, but not restricted to, the health professional degrees. Achieving this remains a test for universities. The moves for registration of the public health workforce globally might also lead to a higher profile for public health career paths, and this could potentially provide more leverage to introduce public health principles and practice into professional entry program curricula.

### Breadth versus depth

A further tension exists between providing the requisite breadth and depth in the understanding of health systems and the place, language and perspectives of the various health disciplines when preparing graduates to be effective "judgement safe" public health practitioners. Getting the balance right is the goal under the current and final phase of PHERP funding where the desired graduates are defined as "having the necessary competencies, including cultural competencies, for public health practice and research, commensurate with national, state and regional public health workforce needs" [16].

PHERP was initially introduced to boost Australia's public health capacity. Building capacity might usefully be considered at three levels:

1. Generic skills in the public health workforce - for example information seeking and synthesis skills, project management, critical appraisal skills, management and leadership.

2. Specialised skills in public health areas where there is a nationally recognised deficit of highly skilled practitioners/researchers - epidemiology, biostatistics, health economics, environmental health and Indigenous health [6].

3. High profile specific strategic needs - specialist skills, and a level of readiness; that is a pool of qualified practitioners that can be mobilised to meet surge capacity needed in the event of sudden impacts - for example unexpected outbreaks such as SARS, pandemic flu and natural and manmade disasters.

The acquisition of the necessary core public health skills and the need for specialisation and expertise to allow graduates to operate independently in their area of interest is a challenge, particularly in those degrees that now seek to achieve this in less than two years full time coursework. This is where other specialist degrees need to be considered for their contribution in bringing essential high level skills into the public health workforce, and where there is a growing need for continuing professional development, for example the Masters of Biostatistics program [17].

The 2005 PHERP Review identified gaps in workforce capacity in areas of specialisation such as indigenous health, epidemiology, health economics and biosecurity [6]. This precipitated some debate on whether educational responses to future workforce needs should shift to target capacity building in specialised skills for emerging health issues or focus on building a robust and responsive generalised workforce. The Review supports a shift in emphasis from university driven education to a more collaborative planning process between government sectors and universities. The intention was for specialised education to be resourced by a contestable PHERP process, but such a funding scheme did not eventuate within PHERP Phase IV.

However, previous PHERP innovation programs have funded curriculum initiatives including a range of distance education resources which do hint at the possibility of national academic institution cooperation in further deepening and broadening public health research and education in Australia. Encouragement of national initiatives that bring together the requisite critical mass of teachers and students for viable teaching programs will foster specialist training and the sharing of limited valuable resources (Indigenous Health educators for example). A national Indigenous public health curriculum framework that sets the standards for Indigenous health content and skills for all public health students18 and a discipline-based public health nutrition initiative built on collegial activities and continuing education links with State health departments http://www.aphnac.com/ are examples of potential templates for collaborations that could coalesce regional or discipline-based curriculum initiatives.

Core public health skills that provide the platform of transferrable knowledge and skills to meet surge capacity demands are required to address both current and emerging national priorities and pandemics. These generic skills are appropriate to the range of emerging public health issues and interventions and, in general, existing university departments of public health are able to respond to this with appropriate support. However, the need to sustain a capacity to respond to current as well as emerging priorities is as much dependant on a flexible workforce allowing mobility in times of response demand as it is on public health graduate attributes.

The working environment of future public health practitioners is unlikely to mirror that of the existing workforce. Protection against public health challenges such as new and emerging infections, terrorism or extreme weather related events demands a strong and innovative workforce capable of rigorous surveillance and research. Educational institutions must therefore focus on emerging trends, and incorporate these into their programs. To this end, universities are increasingly emphasising lifelong learning skills as a key graduate attribute, and graduates who take this ethos (and the necessary skills) into the work place help to build a learning-oriented public health workforce, able to deliver and maintain robust but flexible public health responses.

Australia's future security is dependent on sustained investment in public health and support of regional as well as local capacity to address infectious disease threats [5]. The summer of 2009 brought widespread flooding across parts north- and central-eastern Australia, plus unprecedented bushfires and heatwaves across the south-eastern parts. The immediate health burden was significant, and the recovery phase, to avert health and social problems was protracted. This strained workforce resources, and the contemporaneous timing of these events hampered some aspects of workforce flexibility, such as sharing of capacity between states. In order to meet workforce demand, the small numbers of public health staff worked excess hours, a process that lasted for weeks. The outbreak of 'Swine Flu' that immediately followed further tested capacity in a strained workforce. The likelihood or timing of a recurrence of such devastating events remains unknowable, but are predicted to increase in frequency and cause more severe peaks in surge demand under a changing climate [19].

The prospect of exceeding response capacity, resulting in a system failure, is an unwanted outcome, reminding us of the importance of Australia having access to sufficient numbers of highly skilled and flexible public health practitioners. However increased demand for public health skills is not restricted to calamitous events. The creeping epidemics of obesity and diabetes promise to significantly diminish future health of the Australian population, and burden the acute heath system. Issues such as ageing, substance abuse, sexual health and more, all demand growing public health capacity to achieve a healthy Australia for 2020 and beyond.

### The role of continuing education

Pathways to boost workforce capacity include a constant stream of new graduates and staff development via continuing education. The need for improved processes and systems for human resource development have been identified as essential for enhanced workforce capacity [20] and new concepts and models that align health policy and workforce development are being developed and tested [21,22].

The success of such strategies will require funding, strategic policy alignment and effective partnerships across sectors. Underpinning this is the requirement for an understanding of the imperative that meeting these emerging challenges demands access to a fully prepared public health workforce.

Continuing professional development in the public health context has traditionally meant up-skilling the existing public health workforce, and in the main has been driven by available expertise, ideological, ad-hoc or industry-driven factors. In the current environment of health reform and the shift to a preventive agenda, the demand for continuing professional development in public health and related areas will face unprecedented demands, and not just for the identified public health workforce.

The widespread inclusion of public health knowledge, skills and values across multiple discipline areas will raise new challenges and opportunities for providers of public health education, and calls for more strategic and innovative approaches to up-skilling. The tertiary sector needs to support a range of programs, both specialised and general, to meet the range of short course and professional certificate educational needs, preferably within a flexible model that allows articulation with formal qualifications.

Continuing professional development training partnerships also form an important knowledge exchange platform between academia, government and industry. The interaction can ensure the contemporary relevance of the academic content and skills covered within short courses and filter back to inform public health award program curricula. These collaborations also expose the workforce to academic training that may encourage members of the workforce back into further education to extend their professional capacity.

### Government investment in Public Health Training

While Australian Government support for public health education through PHERP will cease after 2010, other national governments are demonstrating decisive action through public health workforce policy and planning strategies. For example, the United States government through 'Healthy People 2010' strategy has established 14 Public Health Training Centres (PHTC). These Centres are situated in Schools of Public Health and geographically distributed across the country to provide competency-based courses for workforce development through a variety of delivery models. The PHTCs operate as an academic and practice collaborative to promote workforce development [23].

Likewise, New Zealand's national strategy for public health workforce development, Uru Kahikatea, has taken a systematic approach to address workforce development [24]. The objectives of the strategy include actions and targets for workforce policy and planning, public health professional infrastructure, information, policy and research, Maori and Pacific workforce development, supportive workplace cultures, public health career promotion and education and training (by developing generic public health competencies to provide a common framework for professional development). The work plan also includes actions and targets to improve the wider health workforce skills and knowledge of health promotion/public health, and to ensure that public health workforce is included in the wider health workforce information programs and planning.

Future policy and planning for continuing professional development must be inclusive of research on-cost effectiveness for educational interventions. A recent systematic analysis of the cost benefit of continuing professional development in health found no empirical evidence to demonstrate cost-benefit of any professional development. The lack of a cost-benefit finding was attributed to the varying quality of the studies [25], highlighting the need for future investment in quality research into continuing professional development to support evidence-based decision making for policy-makers and contribute to health outcomes for the public.

In Australia, the National Health Workforce Planning and Research Collaboration was established to provide innovation and research to achieve health workforce sustainability by 2020. The Collaboration aims to build capacity in research by improving intellectual and methodological rigour in national health workforce planning, and provide evidence to inform policy decision-making about the health workforce. Regardless of the national focus on prevention, this national body, as its predecessor planning body, the National Health Workforce Taskforce, has excluded the public health workforce from their deliberations and work plan [26].

The Reform Commission has recommended a National Health Promotion and Prevention Agency. It is not clear at this stage where the governance and responsibility for the public health workforce will be positioned within these new arrangements, nor what links there might be to education and training institutions.

### Meeting contemporary public health education challenges

Public health education has traditionally been delivered by universities, the health profession, or as in-house training within the public health workplace. However, initiatives at the TAFE and other registered training organisations that lead to accredited qualifications cannot be ignored. For example, a Certificate IV in Population Health is now being offered through the Adelaide Western General Practice Network (AWGPN). There is already precedent for TAFE awarded degrees (eg engineering). With a shrinking national health workforce and increasing demands that will be placed upon it under the Preventive Health agenda, alliances with this sector need to be considered together with possibilities for work-integrated learning and articulated pathways.

The key concern for public health is the health of populations. Bearing this in mind, what pathways in public health education and training best serve this cause? What should our education priorities in the academic sector be then, and who sets the agenda? Whilst there is divided opinion on whether educational responses to future workforce needs should focus on capacity building in emerging specialised skills sets or a robust and responsive generalised workforce, there is no debate over the need for strong core skills sets across the public health sector. There is also agreement on the need to focus on building capacity in those core discipline areas where we are currently experiencing a recognised capacity deficit (epidemiology, biostatistics, health economics, and environmental and Indigenous health). However specialisation in targeted areas (biosecurity responses etc.) will only be a worthy investment if built on a solid theoretical foundation and skill base, and this is true at both individual public health practitioner and workforce levels. We in the tertiary sector must therefore focus on providing both the underpinning training as well as targeted programs addressing specialisation gaps and emerging special skill needs.

Research higher degree training is a university enterprise, and this is an area that was not addressed under the PHERP agenda and urgently needs attention. Some disciplines are considering strategies to encourage the development of more advanced skills in the workforce (the Biostatistics Consortium of Australia) and to encourage more exceptional students into a research higher degree pathway [27]. However, this is an area that requires more discussion amongst the public health profession, both in terms of building supervision capacity and student project opportunities, and in raising the profile of public health research careers.

The New Zealand Population Health Workforce Plan includes strategies for training providers to strengthen public health skills of the primary care and nursing workforce [27]. Even without such leverage or guidance, many Universities have tried to develop their own solutions, but with mixed success. By popular vote, this will be a future theme for an ANAPHI Teaching and Learning Forum, where representatives across the public health professions and educational institutions come together to share strategies and jointly set the educational agenda for Australia.

## Conclusions

To a certain extent, the emerging political agenda around health in Australia will influence the way public health education will evolve and develop to meet future challenges. Greater collaboration across interest groups and public health disciplines will facilitate and enhance the processes for setting future directions and will shape our success in meeting current and future workforce needs. The Population Health Congress coalition of the four major public health professional associations, scheduled to meet every two to four years, will strengthen the political voice for public health leadership and advocacy. Similarly, the Australian Network of Academic Public Health Institutions, or its post-PHERP successor, will continue to play an important role as a focus for discussions on future educational opportunities, and meeting public health workforce training needs.

There are significant challenges in determining how we chart our way ahead in public health education. We must not lose sight of the fact that much of what we do, particularly in engaging with stakeholders and responding to workforce needs, is exemplary, albeit outside a national or state framework. While this engagement remains ad hoc, there is no way forward for nomenclature and enumeration. Whilst educationalists in other discipline areas look to public health as a model of interdisciplinary and inter-professional education, we do need to build a stronger and more unified public health professional presence and, together, take ownership of setting the public health education agenda. The momentum of the 2020 Summit and valuing of health promotion, further enlivened by the joint resolutions of the first Population Health Congress in July 2008, and the potential that the public health professional coalition represented in the Congress brings, should not be lost.

## Authors' contributions

CB led the development of the focus of the paper through her role as working group chair, drafted the manuscript, and was responsible for coordinating author contributions in the preparation of the final manuscript. KL contributed to the shaping of the paper, and made significant contribution to the sections on continuing education, and current policy/workforce development initiatives. HY contributed to the workshops and discussions that led to the formulation of this paper, and contributed to the drafting of this paper, and the text on undergraduate education in particular. EP contributed to the initial concept of the paper; provided ideas for the section on the public health professional and undergraduate education and worked with KL on editing the penultimate draft. EG provided input at all stages of the discussions that shaped this paper from inception to final draft, and assisted CB in the compilation of the final draft. EH contributed to discussions that shaped this paper and to the drafting of the final manuscript. PR contributed to the sections on the international public health training context, in particular the issues surrounding workforce accreditation. All authors read and approved the final draft.

## Authors' information

All authors are all members of the Australian Network of Public Health Institutions working party formed in 2006 to examine Public Health Quality Agenda and Educational Pathways and are employed in Australian Schools/Departments of Public Health. CB was chair of this working party.

## Declaration of competing interests

The authors declare that they have no competing interests.
